# Red Yeast Rice-Driven Kombucha Fermentation: A Novel Strategy for Developing Functional Beverages with Enhanced Hypoglycemic and Hypolipidemic Properties

**DOI:** 10.3390/foods15040747

**Published:** 2026-02-18

**Authors:** Kai Tong, Yuxue Liao, Yongqing Tang, Yaxin Luo, Xuan Liu, Dan Yu, Jingxuan Zhou, Chenjin Hou, Zhaoling Li

**Affiliations:** 1 School of Food and Liquor Engineering, Sichuan University of Science and Engineering, Yibin 644000, China; tongkai@suse.edu.cn (K.T.); 323083202108@stu.suse.edu.cn (Y.L.); 325095102418@stu.suse.edu.cn (Y.L.); 324083202115@stu.suse.edu.cn (D.Y.); 325095102407@stu.suse.edu.cn (C.H.); 2 Chengdu-Chongqing Economic Circle (Luzhou) Advanced Technology Research Institute, Luzhou 646000, China; tyq_tangyongqing@163.com (Y.T.); liuxuan_lx2025@163.com (X.L.); zhoujinxuan0110@163.com (J.Z.)

**Keywords:** kombucha, black tea, red yeast rice, hypoglycemic activity, hypolipidemic activity, microbial diversity

## Abstract

To address the limited functional diversity of traditional kombucha, this study utilized red yeast rice (RYR) as an alternative substrate and prepared three samples: black tea kombucha (KBT), black tea-red yeast rice mixed kombucha (KBL, at a 1:1 ratio), and red yeast rice kombucha (KRY). After 9 days of fermentation, KRY exhibited the lowest pH, the highest total acidity, and notable sugar metabolic activity. It exhibited in vitro inhibition rates of 82.8%, 78.2%, 70.3%, and 76.9% against cholesterol esterase, pancreatic lipase, α-glucosidase, and α-amylase, respectively, indicating potential hypoglycemic and hypolipidemic activities. In contrast, KBT maintained the strongest antioxidant capacity, with scavenging rates exceeding 90% against both 2,2-diphenyl-1-picrylhydrazyl (DPPH) and 2,2′-Azinobis-(3-ethylbenzthiazoline-6-sulphonate) (ABTS). A total of 72 volatile flavor compounds (VFCs) were identified, with 7 key compounds enriched in KRY, which enhanced its sensory acceptance and received the highest scores in color, clarity, and aroma. Microbial community analysis revealed the post-fermentation dominance of *Komagataeibacter*, Acetobacter, and Saccharomyces, which correlated positively with key VFCs. These findings indicate that RYR as a substrate enhances functional microbial growth, sugar metabolism, organic acid production, flavor enrichment, and in vitro inhibitory activity of enzymes associated with hypoglycemic and hypolipidemic effects.

## 1. Introduction

Kombucha, a traditional acidic tea beverage with a consumption history exceeding 2000 years in Asia, is produced through the symbiotic fermentation of tea infusions and sucrose by symbiotic colony of bacteria and yeast (SCOBY) comprising yeasts, acetic acid bacteria, and lactic acid bacteria [1]. During fermentation, microbial activity generates a range of active compounds, including acetic acid, gluconic acid, glucuronic acid, amino acids, vitamins, and tea polyphenols, which endow kombucha with distinctive organoleptic properties and health benefits, such as antioxidant, anti-inflammatory, and gut microbiota-regulating effects [2,3]. In recent years, driven by growing consumer demand for functional fermented beverages, research on kombucha has shifted toward exploring alternative fermentation substrates to expand its functional diversity and flavor profiles, with reported substrates including grains, teas, fruits, herbs, spices, milk, and by-products from the food industry [2,3]. However, most of these studies focus on auxiliary additions to traditional tea substrates, and there remains a lack of systematic exploration of non-tea functional raw materials as core fermentation substrates for kombucha.

Red yeast rice (RYR), a traditional Chinese medicinal and edible fermented product derived from the solid-state fermentation of rice with *Monascus purpureus*, has garnered significant attention due to its unique bioactive components and functional properties [4,5]. The metabolism of *Monascus purpureus* during fermentation produces a variety of valuable compounds, including natural pigments, monacolins (polyketide derivatives with lipid-lowering activity), rice polysaccharides, and phenolic substances [6,7,8]. These components not only enhance the color and flavor of fermented products but also endow RYR with well-established hypoglycemic, hypolipidemic, and antioxidant properties, making it a widely utilized raw material in the brewing, food processing, and healthcare industries [5]. Despite its documented functional potential, the application of RYR as a primary substrate for kombucha fermentation, along with its effects on kombucha’s physicochemical characteristics, microbial community dynamics, flavor formation, and targeted metabolic regulation, has not been comprehensively investigated.

In this study, we aimed to evaluate the feasibility of RYR as a novel functional substrate for kombucha production by comparing three types of kombucha: black tea kombucha (KBT), black tea-red yeast rice mixed kombucha (KBL, at a 1:1 ratio), and red yeast rice kombucha (KRY). A multi-dimensional analysis was conducted to characterize the dynamic changes in physicochemical indices, bioactive potentials, volatile flavor compounds (VFCs), and microbial community structures. Additionally, we elucidated the correlations between functional microbial taxa and key VFCs to uncover the underlying mechanisms of RYR’s impact on kombucha quality. Overall, this study expands the substrate options for kombucha production, provides theoretical insights into the microbial-driven regulation of functional fermented beverages, and lays a foundation for the development of high-value kombucha products with targeted metabolic health benefits.

## 2. Materials and Methods

### 2.1. Materials and Reagents

The kombucha starter culture was purchased from Zhishi Kombucha, an online retailer based in Shandong Province, China, which specializes in various microbiological starters. Black tea was obtained from a local market in Yibin City, Sichuan Province, China. Red yeast rice was procured from Beijing Tongrentang. Phenol, 2, 2-diphenyl-1-picrylhydrazyl (DPPH), gallic acid, rutin, 3-nonanone, 3,5-dinitro salicylic acid and 2,2′-Azinobis-(3-ethylbenzthiazoline-6-sulphonate) (ABTS) were obtained from Macklin Biochemical Co., Ltd. (Shanghai, China). 4-nitrobenzoic acid ester (p-NPB), 4-nitrophenyl-D-glucopyranoside (PNPG), Pancreatic lipase, α-amylase and α-glucosidase were obtained from Aladdin Reagent Co., Ltd. (Shanghai, China). All other chemicals used in the experiments were of at least analytical grade and were sourced from Xilong Scientific Co., Ltd. in Guangdong, China.

### 2.2. Kombucha-Fermented Production

The kombucha preparation method was slightly modified based on the research by Wang et al. [1]. Specifically, KBT was prepared by infusing 6 g of black tea in 400 mL of boiling water, along with 30 g of sucrose, and steeping at 90 °C for 5 min. After removing the tea leaves, the steeped liquid was transferred to a sterilized glass jar, cooled to room temperature, and inoculated with 20 mL of kombucha liquid and 10 g of SCOBY culture [9]. Additionally, two products were created: KBL, which was made by mixing black tea and red yeast rice in a 1:1 ratio (a blend of 3 g black tea and 3 g red yeast rice), and KRY (6 g red yeast rice), which was prepared using red yeast rice. All samples were incubated at 28 °C for 9 d [10], with physicochemical indicators measured every 3 d. The preparation process for the different types of kombucha is shown in Figure S1.

### 2.3. pH and Total Acidity Analysis

The pH values were measured using a calibrated pH meter. Total acidity was determined following the method described by Dartora et al. [11], utilizing a 0.1 mol/L NaOH solution and phenolphthalein as an indicator.

### 2.4. Sugar Consumption Capacity Analysis

The content of reducing sugars was determined using the 3,5-dinitrosalicylic acid (DNS) method [12]. Reducing sugars were determined using the dinitrosalicylic acid method, with absorbance measurements conducted at 540 nm using a UV spectrophotometer. Total sugars were evaluated through the phenol-sulfuric acid method, in which 0.5 mL of 5% phenol was combined with 1 mL of kombucha, followed by the addition of 2.5 mL of concentrated sulfuric acid. The resulting mixture was heated in boiling water for 15 min before measurement at 490 nm.

### 2.5. Analysis of Microbial Changes

Microbial biomass was monitored throughout the fermentation process by measuring the optical density of samples at 600 nm using a spectrophotometer. Samples were collected on days 0, 3, 6, and 9 for microbial enumeration. Potato dextrose agar (PDA) was employed to culture yeasts, while Man Rogosa and Sharpe (MRS) medium was utilized for culturing lactic acid bacteria. Additionally, glucose yeast extract calcium carbonate agar (GYC) medium was used for the cultivation of acetic acid bacteria [13]. Colonies were counted in triplicate, and the average number of colonies was recorded.

### 2.6. Analysis of Total Polyphenol and Total Flavonoid Content

Total polyphenol content was analyzed using the Folin–Ciocalteu method [14]. Specifically, 6 mL of sample was mixed with 0.5 mL of Folin’s reagent and incubated in the dark for 4 min. Subsequently, 1.5 mL of saturated sodium carbonate solution (75 g/L) was added, and the mixture was incubated in the dark for 30 min. The absorbance was measured at 760 nm, with the total polyphenol content of kombucha quantified as gallic acid equivalents. The total flavonoid content was determined using the nitrite colorimetric method [15]. 10 mL of sample was mixed with 1 mL of 4% sodium nitrite solution and incubated at room temperature for 6 min. Following this, 1 mL of 10% aluminum nitrate solution was added, and the mixture was incubated for 6 min at room temperature. Finally, 8 mL of 4% sodium hydroxide solution was added, and absorbance was measured at 510 nm. The total flavonoid content was quantified as rutin equivalents. The standard curve is shown in Table S1.

### 2.7. Antioxidant Analysis

The DPPH radical scavenging activity was assessed using the method described by Sun et al. [16]. After mixing 2 mL of the sample solution or anhydrous ethanol with 2 mL of a working solution containing 0.2 mM DPPH was incubated in the dark for 30 min. The absorbance of the samples was subsequently measured at 514 nm to determine the scavenging capacity. The ABTS radical cation scavenging ability was evaluated according to the method described by Sun et al. [17]. The ABTS solution was diluted with ethanol to achieve an absorbance of 0.70 (±0.02) at 734 nm and was maintained in the dark for 12 h. Then, 1 mL of the sample solution or anhydrous ethanol was mixed with 3 mL of the working solution containing the ABTS radical, and the absorbance was measured at 734 nm after 6 min. A standard curve for vitamin C equivalent antioxidant capacity (VCEAC) was plotted, the VCEAC of kombucha was calculated based on the curve. The standard curve is shown in Table S1.

The DPPH and ABTS radical scavenging rates were calculated using the following Formula (1):

(1) $$\mathrm{Scavenging}\;\mathrm{rate}\;(\%)\;=\;\left[1\;-\;\frac{\left(A_{2}\;-\;A_{1}\right)}{A_{2}}\right]\times 100\%$$

where A_1_ is the absorbance of the sample group, and A_2_ is the absorbance of the blank group.

### 2.8. Hypolipidemic Activity Analysis

The inhibitory effects of kombucha on pancreatic lipase were assessed using the method described by Huang et al. [18]. In brief, 0.2 mL of kombucha was mixed with 1.0 mL of Tris-HCl (50 mM), 0.4 mL of porcine pancreatic lipase (900 U/mL), and 0.4 mL of 4-nitrobenzoic acid ester (p-NPB) (6 mM). The mixture was incubated at 37 °C for 10 min. After an incubation period of 120 min at 37 °C, and the absorbance was measured at 405 nm.

The cholesterol esterase inhibitory activity of kombucha was determined according to Fangfang et al. [19]. Specifically, 0.2 mL of kombucha was combined with 1.0 mL of sodium phosphate buffer (100 mM) containing 0.2 mL of cholesterol esterase (10 U/mL) and p-NPB (4 mM). After 30 min at 37 °C, the absorbance was measured at 405 nm. The inhibition rates of pancreatic lipase and cholesterol esterase were calculated using Formula (2):

(2) $$\mathrm{Inhibitory}\;\mathrm{rate}\;=\;\left[1\;-\;\frac{\left(A_{1}\;-\;A_{2}\right)}{\left(A_{3}\;-\;A_{4}\right)}\right]\times 100\%$$

where A_1_, A_2_, A_3_, and A_4_ were defined as the absorbance of the sample group, the sample control group, the blank group, and the blank control group, respectively.

### 2.9. Hypoglycemic Activity Analysis

The α-amylase inhibition activity was assessed using the method described by Kaikai et al. [20]. A total of 0.2 mL of kombucha was homogenized with 0.2 mL of α-amylase solution (1 U/mL). The mixture was then incubated at 37 °C for 30 min. Subsequently, 1 mL of 3,5-Dinitrosalicylic acid (DNS) was added, and the absorbance was measured at 540 nm.

The α-glucosidase inhibitory activity of kombucha was evaluated based on the method introduced by Qun et al. [21]. 0.1 mL of kombucha was combined with 0.4 mL of sodium phosphate buffer (100 mM) and 0.2 mL of α-glucosidase solution (1 U/mL); subsequently, 0.2 mL of 4-nitrophenyl-D-glucopyranoside (PNPG) (4 mM) was introduced into the preheated mixture, which was then allowed to react at 37 °C for 30 min before measuring the absorbance at 405 nm. The inhibition rates of α-amylase and α-glucosidase were calculated using Formula (3):

(3) $$\mathrm{Inhibitory}\;\mathrm{rate}\;=\;\left[1\;-\;\frac{\left(A_{1}\;-\;A_{2}\right)}{\left(A_{3}\;-\;A_{4}\right)}\right]\times 100\%$$

where A_1_, A_2_, A_3_, and A_4_ were defined as the absorbance of the sample group, the sample control group, the blank group, and the blank control group, respectively.

### 2.10. Electronic Nose (E-Nose) Analysis

The olfactory profiles of kombucha samples were evaluated using a PEN3 E-nose system (Shanghai Angshen Intelligent Technology Co., Ltd., Shanghai, China). A precise volume of 8 mL from each sample was measured, sealed in a 20 mL glass vial, and subsequently placed in a water bath at 50 °C for 5 min prior to testing. The sample preparation duration was 10 s, while the sensor cleaning period lasted for 90 s. The injection flow rate was maintained at 1.0 L/min, and data collection was performed over a duration of 60 s. The system was reset and standardized before subsequent headspace sampling [22]. The characteristics of the E-nose sensors are shown in Table S2.

### 2.11. Electronic Tongue (E-Tongue) Analysis

The taste characteristics of kombucha were analyzed using an E-tongue system (Shanghai Angshen Intelligent Technology Co., Ltd., Shanghai, China). To prepare the samples, 40 mL of diluted kombucha was accurately pipetted into designated measuring cups, with the sensor immersed in each sample for 30 s. The experiments were conducted at room temperature, and the detection probes were thoroughly cleaned with distilled water both before and after each test [23]. All analyses were performed in triplicate.

### 2.12. Sensory Evaluation

The sensory evaluation was conducted by a trained panel consisting of 10 experienced members. These panelists underwent a comprehensive training program aimed at enhancing their familiarity with the aroma characteristics of the kombucha samples. The kombucha was assessed across five dimensions: color, sweet and sour taste, wine aroma, tea aroma, and overall acceptability. A 9-point scale was employed to evaluate sensory intensity, where scores of 0–1 indicated weak intensity, 2–3 indicated low intensity, 4–5 indicated moderate intensity, 6–7 indicated high intensity, and 8–9 indicated the highest intensity. Subsequently, the panelists evaluated the kombucha samples and recorded the aroma intensity for each sensory descriptor, with each sample being assessed in triplicate [24]. This study was approved by the Ethics Committee of Sichuan University of Science and Engineering (approval number: 2025LLSC023), and all participants signed informed consent forms.

### 2.13. HS-SPME-GC-MS Analysis

The measurement of VFCs in kombucha was performed according to the method previously reported by Huang et al. [25], with slight modifications. Kombucha samples were stored at 4 °C prior to headspace solid-phase micro-extraction (HS-SPME). An 8 mL sample of kombucha, along with 20 μL of an internal standard (3-nonanone, 9.8 mg/L) and 1 g of NaCl, was placed in a 20 mL glass vial. After equilibrating and stabilizing at 50 °C for 5 min, the SPME fiber was employed to absorb VFCs for 30 min. Following absorption, the VFCs were desorbed in a gas chromatography–mass spectrometry (GC–MS) injector at 220 °C for 5 min. Helium was utilized as the carrier gas at a flow rate of 1 mL/min. The samples were analyzed using a DB-5MS UI column (30 m in length, 0.250 mm in inside diameter, and 0.25 μm in film thickness, Agilent). The column temperature was programmed as follows: an initial temperature of 35 °C held for 5 min, increased at a rate of 4 °C/min to 130 °C and held for 3 min, followed by an increase at 5 °C/min to 230 °C, held for an additional 5 min. Electron impact ionization was conducted at 70 eV, scanning from m/z 10 to 280. Background subtraction was performed on the raw GC–MS data using data processing software (Agilent Mass-Hunter Qualitative Analysis 12.0). Compound identification was achieved by matching the mass spectra with the NIST14 mass spectral database. The relative area percentages of the VFCs (relative to the total peak area) were exported from the database. Concentration values were calculated based on the ratio of peak areas between the internal standard and the volatile compounds.

### 2.14. Microbial Community Diversity

The microbial genomic DNA from the samples was extracted using the FastPure Microbiome DNA Isolation Kit (Omega Bio-tek, Norcross, GA, USA). The concentration and purity of the DNA were assessed via 1% agarose gel electrophoresis. The DNA concentration was then diluted to 1 μg/μL using sterile water, with 10 ng of the diluted DNA serving as the template. For bacteria analysis, the V3–V4 hypervariable regions of the 16S rRNA gene were amplified using the universal primer sequences 338F (5′-ACTCCTACGGGAGGCAGCAG-3′) and 806R (5′-GGACTACHVGGGTWTCTAAT-3′). For fungal analysis, the internal transcribed spacer regions were amplified using the universal primer sequences ITS1 (5′-CTTGGTCATTTAGAGGAAGTAA-3′) and ITS2 (5′-GCTGCGTTCTTCATCGATGC-3′). The PCR amplification cycling conditions were as follows: an initial denaturation at 95 °C for 3 min, followed by 27 cycles of denaturation at 95 °C for 30 s, annealing at 55 °C for 30 s, and extension at 72 °C for 45 s, concluding with a single extension at 72 °C for 10 min, and ending at 4 °C. Equal volumes of purified amplicons were combined and subjected to paired-end sequencing on an Illumina Nextseq2000 platform (Illumina, San Diego, CA, USA), following the standard protocols provided by Majorbio Bio-Pharm Technology Co., Ltd. (Shanghai, China). Subsequently, chimeric sequences were removed, and low-quality sequences were excluded to obtain high-quality sequences suitable for precise analysis [26].

### 2.15. Statistical Analysis

The data were analyzed using SPSS version 18.0 (IBM, Armonk, NY, USA) and presented as means ± standard deviation. The statistical significance of differences between groups was assessed using Duncan’s multiple-range test, with a *p*-value of less than 0.05 considered indicative of significant differences. Graphs and figures were generated using Origin 2024 (OriginLab, Massachusetts, MA, USA). Partial Least Squares Discriminant Analysis (PLS-DA) and Orthogonal Partial Least Squares Discriminant Analysis (OPLS-DA) models were constructed with SIMCA version 14.1 (Umetrics AB, Umeå, Västerbotten, Sweden). The heatmap was created using TBtools version 1.1.08. Microbiota bioinformatics analysis was conducted via the Majorbio cloud platform (https://cloud.majorbio.com). All experiments were performed in triplicate.

## 3. Results and Discussion

### 3.1. Appearance of Kombucha During Fermentation

**Figure 1:**
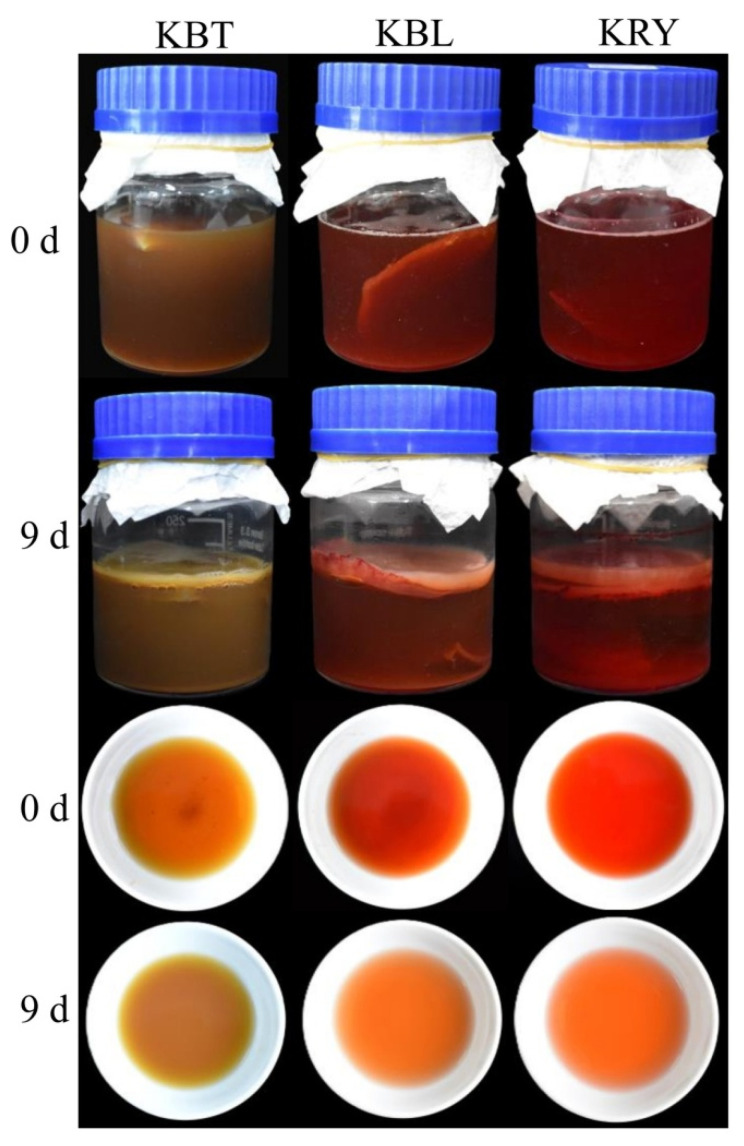
The changes in appearance characteristics of KBT, KBL, and KRY during the fermentation.

Due to the inherent characteristics of the raw materials, kombucha exhibits a variety of colors. As shown in Figure 1, KBT presents a brown color, while KBL and KRY display a reddish hue during the initial stages of fermentation. In KBT, catechins undergo oxidation, transforming into reddish-orange pigments and theaflavins under the catalysis of oxidase, resulting in a darker color after infusion [27]. Conversely, the red pigments produced by *Monascus* fermentation are soluble in the liquids of KBL and KRY. Following 9 d of fermentation, the color of the tea liquid lightens, and a thick bacterial cellulose membrane forms on the surface [28]. As shown in Figure 1, the bacterial cellulose membrane produced in KBL and KRY is thicker than that in KBT. The bacterial cellulose membrane consists of cellulose generated by acetic acid bacteria, while the ethanol produced by yeast promotes the growth of these bacteria, leading to the production of additional bacterial cellulose and acetic acid [29]. These findings suggest that incorporating red yeast rice as a fermentation substrate for kombucha may enhance the proliferation of yeast and acetic acid bacteria, resulting in increased production of bacterial cellulose.

### 3.2. pH and Total Acidity Changes During Fermentation

**Figure 2:**
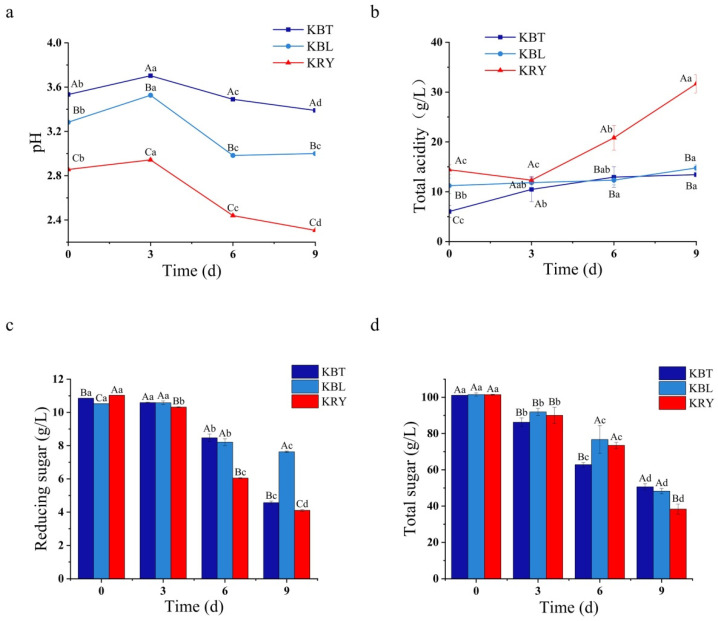
The variations in pH (**a**), total acidity (**b**), reducing sugar (**c**), and total sugar (**d**) during the fermentation of KBT, KBL, and KRY, with equal lowercase letters indicating no significant differences between fermentation days and equal capital letters indicating no significant differences between samples.

The pH value and total acidity significantly influence microbial activity and the stability of metabolites in kombucha, while simultaneously inhibiting the growth of competing bacteria [30]. As shown in Figure 2a, the initial pH values for KBT, KBL, and KRY were recorded at 3.53, 3.28, and 2.86, respectively. After 9 d of fermentation, these pH values decreased to 3.39, 3.00, and 2.31. Correspondingly, the initial total acidity values for KBT, KBL, and KRY were 6.04 g/L, 11.21 g/L, and 14.42 g/L, respectively (Figure 2b). After 9 d of fermentation, the total acidity values increased by 122.35%, 31.94%, and 119.63%, respectively (Figure 2b). The observed decrease in pH and the increase in total acidity can be attributed to the organic acids produced during microbial metabolism. During the fermentation process of kombucha, yeast converts sugars into ethanol and carbon dioxide via the glycolysis pathway [31], while acetic acid bacteria further convert ethanol into acetic acid and tartaric acid. Furthermore, with the extension of fermentation time, organic acids such as malic acid and succinic acid are also produced. These organic acids not only contribute to the sour taste but also serve as precursors for various VFCs.

### 3.3. Sugar Consumption During Fermentation

Sugar is extensively employed as the carbon source for kombucha fermentation, serving as the primary energy source for microorganisms. It is metabolized into various small molecules, including organic acids, phenolics, and amino acids [32]. As shown in Figure 2c, during the initial fermentation period (0–3 d), the reducing sugar content in KBT, KBL, and KRY was approximately 11 g/L. After 9 d of fermentation, the reducing sugar content in KBT and KRY significantly decreased, with reductions of 57.88% and 62.74%, respectively, compared to 0 d. In contrast, the reducing sugar content in KBL exhibited a decrease of only 27.54%. These findings suggest that microorganisms adapt to the new environment during the initial fermentation stage; as fermentation progresses, acetic acid bacteria gradually multiply, oxidizing ethanol into acetic acid or other organic acids [33]. After 9 days of fermentation, KBL displayed a higher content of reducing sugars, likely due to its lower population of acetic acid bacteria, resulting in a reduced conversion of reducing sugars. Correspondingly, the total sugar content of KBT, KBL, and KRY exhibited a declining trend (Figure 2d). Initially, the total sugar contents in KBT, KBL, and KRY were 101.12 g/L, 101.52 g/L, and 101.44 g/L, respectively. After 9 d of fermentation, the total sugar content in the respective fermentation broths was 50.62 g/L, 48.26 g/L, and 38.33 g/L, representing reductions of 49.94%, 52.46%, and 62.21%, compared to the initial stage. The significant decrease in total sugar levels can be attributed to the substantial proliferation of yeast during the fermentation process, which consumed a considerable amount of sugar, leading to a rapid decline in total sugar content [34].

### 3.4. Changes in Microbial Characteristics During the Fermentation of Kombucha

**Figure 3:**
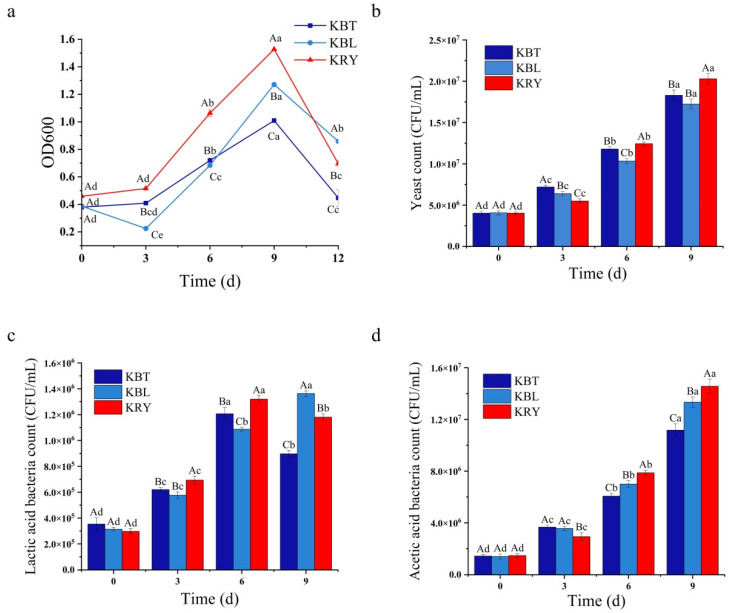
The changes in biomass (OD600) (**a**), yeast colonies (**b**), lactic acid bacteria colonies (**c**), and acetic acid bacteria colonies (**d**) changes in KBT, KBL, and KRY during fermentation, with equal lowercase letters indicating no significant differences between fermentation days and equal capital letters indicating no significant differences between samples.

The optical density at 600 nm (OD600) was utilized to evaluate microbial growth throughout the fermentation process [25]. As shown in Figure 3a, the OD600 values for KBT, KBL, and KRY ranged from 0.38 to 0.46 during the initial fermentation. After 9 d of fermentation, the OD600 values for KBT, KBL, and KRY peaked, with KRY exhibiting the highest OD600 value of 1.53, indicating a more substantial microbial population. However, as the fermentation period extended to 12 d, the production of organic acids, mainly acetic acid by acetic and lactic acid bacteria, significantly inhibited microbial growth, leading to a decline in the number of viable bacteria during the later stages of fermentation [35]. These findings indicated that different substrates significantly influence microbial growth during kombucha fermentation.

Kombucha is a fermented beverage produced through a symbiotic relationship among acetic acid bacteria, lactic acid bacteria and yeast. The counts of these strains significantly influence the chemical composition and flavor substances of kombucha. During the initial fermentation stage, the counts of yeast, lactic acid bacteria, and acetic acid bacteria in KBT, KBL, and KRY were relatively low, with no significant differences observed among these samples (Figure 3b–d). As fermentation progressed, the counts of these microorganisms gradually increased. After 9 d of fermentation, the yeast count in KRY reached 2.03 × 10^7^ CFU/mL, which was significantly higher than that in KBT (1.83 × 10^7^ CFU/mL) and KBL (1.72 × 10^7^ CFU/mL) (*p* < 0.05) (Figure 3b). Yeast has long been utilized in food fermentation due to its ability to hydrolyze various substrates and metabolize monosaccharides to synthesize specific esters, thereby enhancing the sensory characteristics and flavor quality of foods [36,37].

The count of lactic acid bacteria in KBT and KRY initially increased, peaking on the 6th d of fermentation at 1.21 × 10^6^ CFU/mL and 1.32 × 10^6^ CFU/mL, respectively, before gradually declining (Figure 3c). This decline can be attributed to the accumulation of lactic and acetic acids, which inhibit the growth of lactic acid bacteria [13]. In contrast, the count of lactic acid bacteria in KBL exhibited a gradual increasing trend, reaching 1.36 × 10^6^ CFU/mL after 9 d of fermentation, with no significant difference compared to the highest count in KRY (Figure 3c). These results suggest that the growth and reproduction of lactic acid bacteria in KBL were slower than in KRY. Additionally, the count of acetic acid bacteria in KBT, KBL, and KRY increased rapidly, reaching 1.11 × 10^7^ CFU/mL, 1.33 × 10^7^ CFU/mL, and 1.46 × 10^7^ CFU/mL, respectively (*p* < 0.05) (Figure 3d). Notably, the count of acetic acid bacteria in KBL and KRY was significantly higher than that in KBT. Given that acetic acid bacteria contribute to the formation of SCOBY in kombucha, this may explain the thicker bacterial cellulose membrane produced in KBL and KRY [29,38].

### 3.5. Determination of Total Polyphenols and Total Flavonoids in Kombucha

As shown in Table 1, the total polyphenol content in KBT and KBL exhibited a decreasing trend with prolonged fermentation time. In contrast, the total polyphenol content in KRY exhibited a trend of increasing with fermentation duration, although it remained significantly lower than that of the other two groups. Additionally, the total flavonoid content in KBT, KBL, and KRY showed a declining trend as fermentation time extended. Polyphenols and flavonoids are primary bioactive components in tea leaves [39], which may explain why KBT and KBL exhibited significantly higher total polyphenol and flavonoid levels than KRY. Furthermore, microorganisms in kombucha can produce or transform small-molecule phenolic compounds through metabolic activity and hydrolytic enzymes, thereby altering total polyphenol and flavonoid content [40], potentially contributing to the observed decreasing trend with prolonged fermentation time.

### 3.6. Antioxidant Activity Evaluation of Kombucha

The antioxidant activity was assessed by measuring the DPPH and ABTS radical scavenging capacities of kombucha samples [37]. As shown in Table 2, KBT displayed the highest DPPH and ABTS equivalent antioxidant capacities, ranging from 1.2761 to 1.2846 mg/mL and 2.0757–2.1311 mg/mL, respectively. Furthermore, the antioxidant potential of KBL, expressed as a percentage of DPPH and ABTS radical inhibition, ranged from 1.1852 to 1.2687 mg/mL and 1.2069–1.4817 mg/mL (Table 2). Conversely, KRY exhibited the lowest antioxidant activity, with inhibition rates of 0.3412–0.4773 mg/mL for DPPH and 0.3870–0.4917 mg/mL for ABTS (Table 2). These findings indicate that black tea possesses superior antioxidant properties compared to red yeast rice, which can be attributed to the rich content of its natural tea polyphenol and flavonoid components [41]. This suggests that KBT is well-suited for development into a daily ready-to-drink kombucha beverage, characterized by a pleasant taste and strong antioxidant capacity, thereby fulfilling the requirements for daily health maintenance.

### 3.7. In Vitro Hypolipidemic Activity

**Figure 4:**
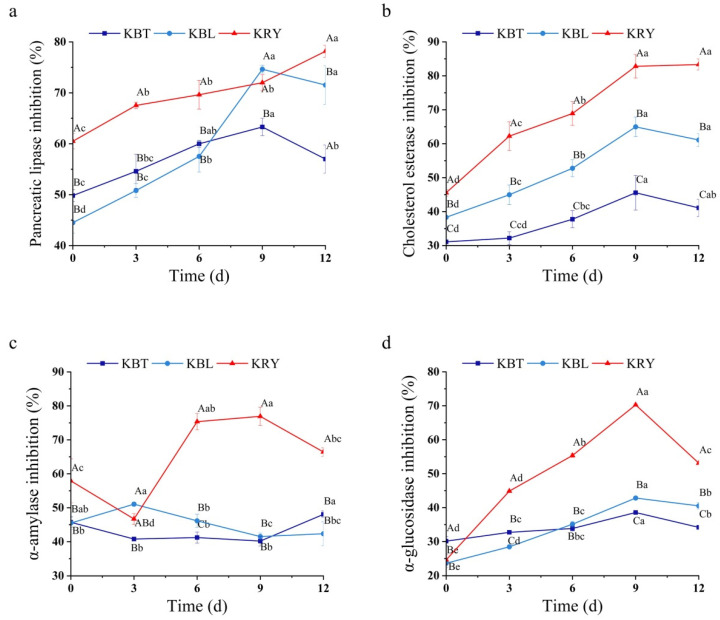
The changes in pancreatic lipase (**a**), cholesterol esterase (**b**), α-amylase (**c**), and α-glucosidase (**d**) inhibition rate changes in KBT, KBL, and KRY during fermentation, with equal lowercase letters indicating no significant differences between fermentation days and equal capital letters indicating no significant differences between samples.

Pancreatic lipase and cholesterol esterase are crucial digestive enzymes that play a pivotal role in regulating lipid digestion and absorption. They are widely recognized as essential therapeutic targets for ameliorating obesity and other lipid metabolism disorders [42]. This study systematically assessed the in vitro inhibitory capacities of KBT, KBL, and KRY on the activities of pancreatic lipase and cholesterol esterase. As shown in Figure 4a,b, the inhibition rates of KBT, KBL, and KRY on pancreatic lipase exhibited a gradual increase with extended fermentation duration. The inhibition rates of KBT and KBL peaked on the 9th day of fermentation, reaching values of 63.3% and 74.6%, respectively, while KRY achieved its highest inhibition rate of 78.2% on the 12th day (Figure 4a). Additionally, KBT, KBL, and KRY displayed similar trends in inhibiting cholesterol esterase, characterized by initially increasing and subsequently decreasing, with peak inhibitory activity observed after 9 days of fermentation. Specifically, the cholesterol esterase inhibition rate of KBT increased from 31.1% to 45.6%, that of KBL from 38.3% to 65%, and that of KRY from 45.6% to 82.3% (Figure 4b). These findings indicate that the fermentation process significantly enhanced the in vitro inhibitory activity of kombucha on pancreatic lipase and cholesterol esterase, likely due to the production of bioactive components such as monascus pigments, fatty acids, sterols, isoflavones, and polyketides during the fermentation of red yeast rice [6,43]. Therefore, KRY has the potential to be developed into a functional special dietary food with hypoglycemic and hypolipidemic effects.

### 3.8. In Vitro Hypoglycemic Activity Evaluation

In human dietary intake, carbohydrates are initially broken down into oligosaccharides by α-amylase and subsequently hydrolyzed into glucose for absorption through the action of α-glucosidase. Consequently, inhibiting the activities of both α-amylase and α-glucosidase can mitigate fluctuations in blood glucose levels, thereby reducing postprandial hyperglycemia, which provides a potential experimental basis for exploring interventions to alleviate postprandial glycemic excursions associated with type 2 diabetes mellitus (T2DM) [8,44,45]. In this study, KBT and KRY exhibited an initial decline followed by an increase in their inhibitory effects on α-amylase activity, whereas KBL displayed an initial increase followed by a decrease (Figure 4c). Notably, after 9 d of fermentation, KRY achieved an α-amylase inhibition rate of 76.9%, significantly higher than those of KBT and KBL (*p* < 0.05). Additionally, the inhibition rates of KBT, KBL, and KRY on α-glucosidase activity exhibited an initial increase followed by a decrease, with KRY demonstrating the strongest inhibitory activity against α-glucosidase, achieving an inhibition rate of 70.3% (*p* < 0.05) (Figure 4d). These results indicate that kombucha fermented with red yeast rice as the substrate exhibits potent inhibitory activity against both α-amylase and α-glucosidase. This bioactivity may be related to the bioactive components characteristic of red yeast rice, as supported by relevant literature reports, while the specific functional metabolites remain to be identified [7,8].

### 3.9. E-Tongue and Nose Analysis and Sensory Evaluation of Kombucha

**Figure 5:**
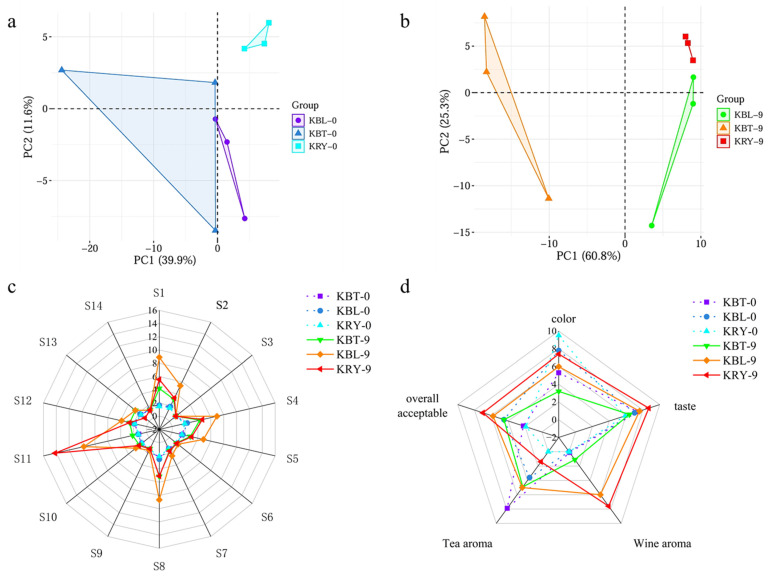
E-tongue (**a**,**b**), E-nose sensor (**c**), and sensory characteristics (**d**) for KBT, KBL, and KRY.

The E-tongue simulates human taste perception to analyze, identify, and evaluate samples, providing a rapid assessment of their taste characteristics [46]. Principal component analysis (PCA), a statistical methodology, is utilized to extract information from variables and elucidate the variations present within the samples [22]. To enhance the visualization of electronic tongue detection results for KBT, KBL, and KRY, PCA was conducted. During the initial fermentation stage, the two primary variance components accounted for a total of 51.5% (Figure 5a). Notably, after 9 d of fermentation, PC1 (60.8%) and PC2 (25.3%) together explained 86.1% of the total variance, suggesting that these components represent the major taste information of kombucha samples (Figure 5b). As fermentation time increased, a distinct separation was observed. These findings indicate that the three types of kombucha exhibit significant differences in taste, and the E-tongue response sensors are proficient at facilitating the rapid identification of kombucha based on their taste characteristics across varying fermentation durations and types.

The E-nose effectively facilitates the non-destructive discrimination of flavor, addressing the inherent subjectivity in human perception [47]. In this study, the E-nose was employed to analyze KBT, KBL, and KRY at 0 and 9 d of fermentation. The response values from the 14 sensors (S1-S14) exhibited similar trends in the radar fingerprint, although the signal intensities varied. As shown in Figure 5c, the signal intensities of the sensors increased with the duration of kombucha fermentation. After 9 d of fermentation, the response values of sensors S1, S2, S4, S5, S8, and S11 in KBT, KBL, and KRY exhibited an upward trend. Notably, the response values of sensor S11 (aromatic compounds) in both KRY and KBL were higher than those in KBT, indicating that the addition of red yeast rice as a fermentation substrate for kombucha enhances the production of aromatic compounds. *Monascus* can produce various hydrolases and transferases including proteases, amylases, and esterases, which are capable of mediating saccharification and esterification reactions that generate flavor-active compounds such as isoamyl lactate and ethyl acetate. The distinctive aroma and improved flavor profile of red yeast rice kombucha may be related to the potential catalytic effects of these enzymes derived from *Monascus*, though direct evidence for this metabolic activity within the fermentation system remains to be confirmed [36,48,49].

The sensory characteristics of three distinct kombucha samples were assessed, focusing on color, taste, wine aroma, tea aroma, and overall acceptability. As shown in Figure 5d, KRY achieved the highest score for appearance, attributable to its appealing color and enhanced transparency. Conversely, KBT received the lowest appearance score due to its turbidity and dark coloration (Figure 1). Following fermentation, KRY’s overall acceptability score was significantly higher than those of KBT and KBL, particularly regarding wine aroma and taste. However, wine aroma is not solely determined by ethanol; the concentration of esters can also produce similar aromas [50]. Certain bacteria can synthesize esters (e.g., acetic acid bacteria synthesizing ethyl acetate), thereby influencing aroma intensity [48]. Therefore, this aroma enhancement may stem from the richer microbial community observed in KRY.

### 3.10. VFCs of Kombucha

**Figure 6:**
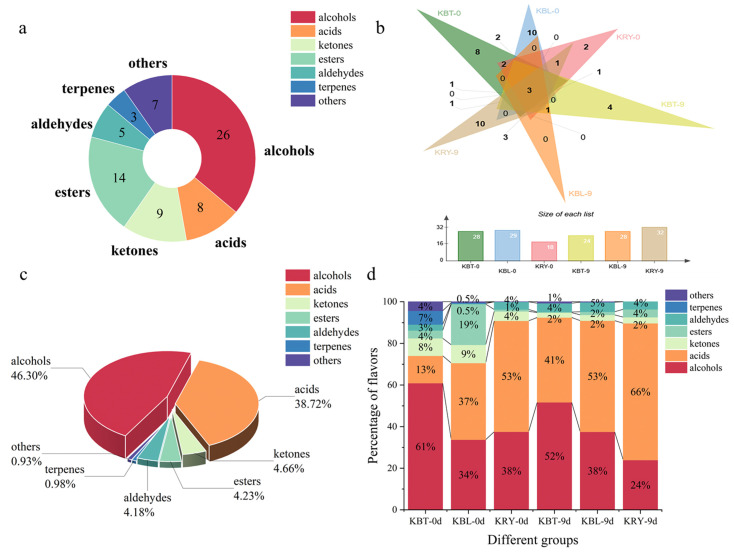
Compares the VFCs profiles from KBT, KBL, and KRY, detailing types of VFCs (**a**), a Venn plot (**b**), percentage of VFCs (**c**), and relative proportion of VFCs (**d**).

VFCs play a crucial role in determining the quality of fermented foods [51]. A total of 72 VFCs were detected in fermented kombucha using HS-SPME-GC-MS, comprising 14 esters, 3 alkenes, 8 acids, 5 aldehydes, 9 ketones, 26 alcohols, and 7 other compounds (Figure 6a and Table S3). During the initial fermentation stage, 28, 29, and 18 VFCs were identified in KBT, KBL, and KRY, respectively (Figure 6b). Notably, after 9 d of fermentation, 32 VFCs were identified in KRY, while 24 and 28 were detected in KBT and KBL, respectively (Figure 6b). Among these volatile compounds, alcohols and acids represented the highest proportions, at 46.30% and 38.72%, respectively (Figure 6c). Specifically, in KBT, the proportion of alcohols was 61% before fermentation, decreasing to 52% post-fermentation, while the proportion of acids increased from 13% to 41% (Figure 6d). Before fermentation, the primary VFCs in KBL were alcohols, acids, and esters, accounting for 34%, 37%, and 19%, respectively (Figure 6d). After fermentation, the proportions of acids and alcohols increased to 53% and 38%, respectively (Figure 6d). For KRY, the proportions of alcohols and acids prior to fermentation were 38% and 53%, respectively. After fermentation, the proportion of alcohols decreased to 24%, while the proportion of acids increased to 66% (Figure 6d). Throughout the traditional kombucha fermentation process, the proportion of alcohol compounds gradually decreased due to microbial transformation, esterification, and other biochemical reactions [52].

**Figure 7:**
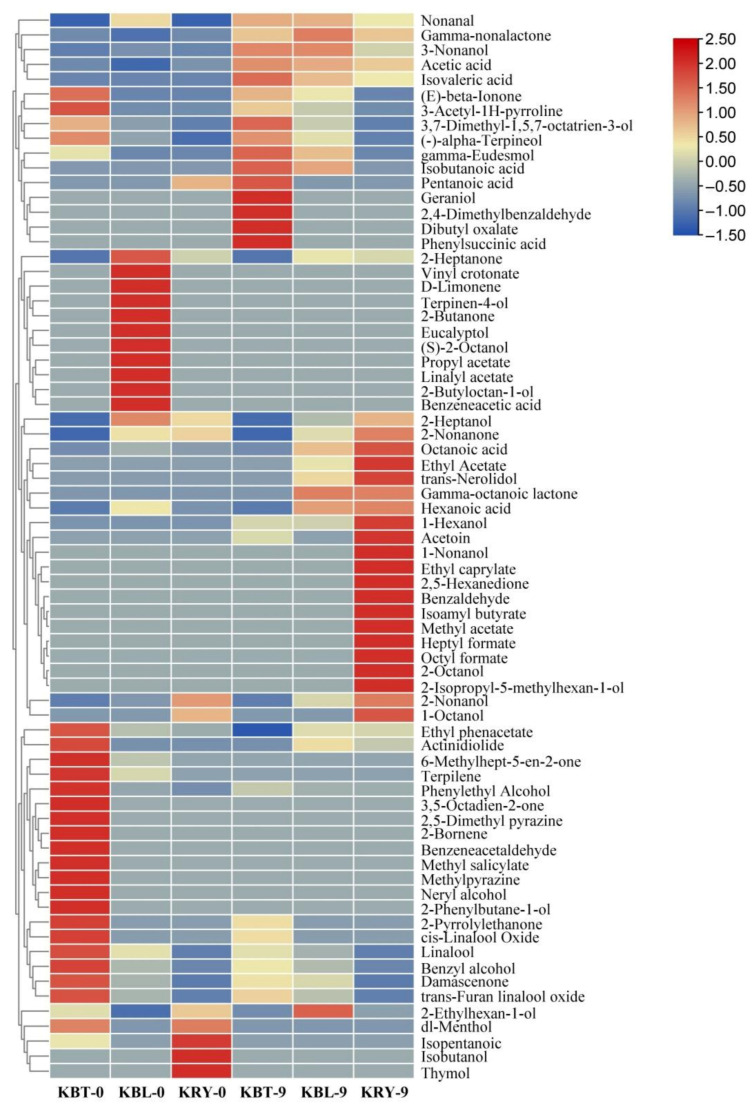
Clustering heatmap illustrating the classification of VFCs among KBT, KBL, and KRY.

A cluster heatmap of VFCs in KBT, KBL, and KRY is shown in Figure 7. The results of the horizontal clustering revealed that all volatile compounds were categorized into four distinct groups. The first group primarily consisted of acids and alcohols, which were found at elevated levels in KBT, underscoring their potential significance in differentiating KBL from other types of kombucha. These compounds included geraniol, 2,4-dimethylbenzaldehyde, dibutyl oxalate and phenylsuccinic acid. The third group, primarily consisting of esters and alcohols, included ethyl acetate, trans-nerolidol, ethyl caprylate, octyl formate, and 2-octanol, which accumulated in KRY after 9 d of fermentation. The VFCs in the second and fourth groups were present at elevated levels only during the initial stage of fermentation in KBL and KBT, respectively.

PLS-DA is a supervised multivariate statistical method utilized to model the regression relationship between multiple independent and dependent variables [53]. Based on fermentation time, seven kombucha samples, including quality control (QC) samples, were discriminated using PLS-DA (Figure S2a). Kombucha fermented for 9 d was positioned on the left side of the *Y*-axis, whereas kombucha fermented for 0 d was located on the right side, with clear distinctions observed among the various samples. Furthermore, the results of 200 permutation tests indicated that the Y-intercept criteria of R^2^ (cum) and Q^2^ (cum) were 0.285 and −0.72, respectively. These findings suggested that the constructed model did not exhibit overfitting, thereby confirming the effectiveness of the model validation (Figure S2b). The PLS-DA model identified 16 VFCs with a Variable Importance Projection (VIP) greater than 1 (Figure S2c). To effectively distinguish the VFCs among samples, an OPLS-DA model was constructed using pairwise comparisons (Figure S3). The explanatory rates (R^2^X, R^2^Y) of all OPLS-DA models exceeded 80%, and the goodness of fit of the models was confirmed through 200 permutation tests (Figure S4). On this basis, the VIP value was established to quantify the contribution level of variables. VFCs with VIP values greater than 1 were identified as potential key aroma compounds, with higher VIP values indicating a greater ability to differentiate sample flavors [54]. Therefore, the OPLS-DA approach effectively identifies and screens key variables as potential key aroma compounds for distinguishing samples (Figure S3). In this study, 19 key VFCs with a VIP greater than 1 were identified: (S)-2-octanol, benzyl alcohol, 2-isopropyl-5-methylhexan-1-ol, trans-nerolidol, 3-nonanol, phenylethyl alcohol, 3,7-dimethyl-1,5,7-octatrien-3-ol, hexanoic acid, acetic acid, octanoic acid, pentanoic acid, isovaleric acid, isobutanoic acid, ethyl caprylate, propyl acetate, ethyl acetate, (E)-β-ionone, acetoin, γ-nonalactone (Figure S5). These compounds contributed to fruity, sweet, floral, citrus, cheesy, creamy, and other aromatic characteristics of kombuchas (Table S4). Notably, 7 key VFCs exhibited high accumulation in KRY, including acetoin, ethyl caprylate, 2-isopropy1-5-methylhexan-1-ol, hexanoic acid, octanoic acid, ethyl acetate, and trans-nerolidol (Figure S5). Acetoin, which is naturally found in foods, possesses a pleasant yogurt-like scent and a creamy butter taste (Table S4) [55]. Furthermore, the proportion of esters was relatively high, playing a vital role in the flavor profile of kombucha. Hexanoic acid and octanoic acid serve as important precursor substances for esters, which may explain the elevated content of ester components in KRY [56].

### 3.11. Microbial Diversity of Kombucha

**Figure 8:**
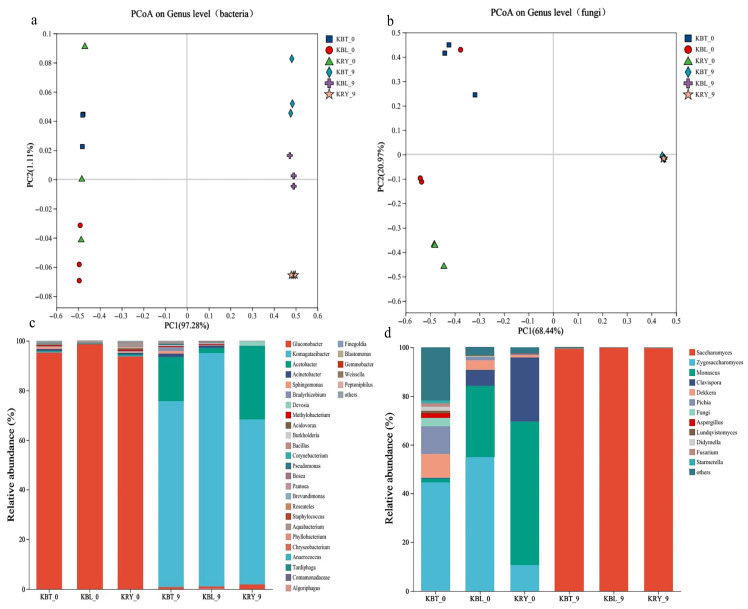
The microbial diversity of KBT, KBL, and KRY, including PCoA analyses of the bacteria community (**a**) and fungal community (**b**), as well as relative abundance (%) of kombucha bacteria (**c**), and fungi (**d**) at the genus level during different fermentation stages.

The differences in microbial communities during fermentation are the primary reason for the notable variations in the sensory quality of fermented foods [49]. To elucidate the microbial composition of KBT, KBL, and KRY, we employed ITS and 16S rRNA gene sequencing techniques. The sequencing coverage for all samples exceeded 99.99%, indicating that the results accurately reflect the microbial community composition in the three kombucha samples. Principal coordinates analysis (PCoA) was employed to investigate the differences in microbial community structures among the various samples. The contribution rates of bacterial principal coordinates PC1 and PC2 were determined to be 98.39%, while those for the fungal principal coordinates PC1 and PC2 were found to be 89.41%. Collectively, these findings elucidate a significant portion of the information inherent in the samples [57]. The similarity in species composition structures correlates with the proximity of distances between samples; thus, samples exhibiting closely related community structures tend to cluster together, whereas those with pronounced community differences are positioned further apart [58]. As shown in Figure 8a,b, the pre-fermentation and post-fermentation samples are distinctly separated, indicating substantial differences in microbial communities before and after fermentation. In contrast, the fungal community structures across all samples exhibited a high degree of similarity following 9 d of fermentation (Figure 8b).

Microorganisms with a relative abundance of ≥1% are classified as dominant flora [59]. A total of 30 bacterial genera and 12 fungal genera were identified in KBT, KBL, and KRY. Mixed fermentation enhances the production of esters (such as ethyl acetate, hexyl acetate, and isobutyl acetate), alcohols (including octanol, 2-methyl-1-butanol, and pentanol), ketones (such as 2-hexanone and 3,4-dimethyl-2-pentanone), and other flavor compounds that contribute floral and fruity aromas reminiscent of banana, apple, and rose [60,61]. As shown in Figure 8c, during the initial stage of fermentation, the bacterial community structure in KBT, KBL, and KRY was predominantly composed of *Gluconobacter*, with an average abundance of 95.82%, followed by *Komagataeibacter*, *Acidovorax*, *Acinetobacter*, and *Sphingomonas*. After 9 d of fermentation, significant changes were observed in the bacterial community structure, characterized by a sharp decrease in *Gluconobacter* across all samples, which accounted for only 1.2% on average. In contrast, *Komagataeibacter* increased dramatically, constituting 74.8%, 93.9%, and 66.4% in KBT, KBL, and KRY, respectively. This shift may be attributed to the strong acid resistance of *Komagataeibacter* [62]. The bacterial flora during kombucha fermentation primarily includes *Gluconobacter*, *Komagataeibacter*, and *Acinetobacter* [63]. Notably, *Komagataeibacter* is closely associated with the production of SCOBY [30]. In KBL, *Komagataeibacter* accounts for the highest proportion, suggesting that the composition of this raw material may promote SCOBY production. Additionally, genera such as *Gluconobacter*, *Komagataeibacter*, and *Acinetobacter* facilitate not only the production of SCOBY but also the accumulation of organic acids, including lactic acid, gluconic acid, and glucuronic acid [64]. Furthermore, *Acetobacter* constitutes a relatively high proportion in KBL and KRY, at 17.73% and 29.6%, respectively.

As shown in Figure 8d, significant differences in fungal colony structures were observed among KBT, KBL, and KRY during the initial stage of fermentation. In KBT, *Zygosaccharomyces* constituted the highest proportion at 44.6%, followed by *Pichia* and *Dekkera* at 11.2% and 9.8%, respectively. In KBL, *Zygosaccharomyces* represented 54.9%, while *Monascus* and *Cladosporium* accounted for 29.3% and 6.5%, respectively. In KRY, *Monascus* was the dominant species, comprising 59.1%, with *Cladosporium* and *Zygosaccharomyces* at 26.3% and 10.5%, respectively. *Monascus* is a vital microorganism recognized for the production of edible natural pigments. *Monascus* pigments (MPs) serve as natural food colorants that not only enhance the visual appeal of foods but also possess antioxidant properties. Additionally, the fermentation state of *Monascus* plays a crucial role in improving the flavor quality of Tartary buckwheat [65]. After 9 d of fermentation, Saccharomyces emerged as the dominant microbial community in KBT, KBL, and KRY, with an average proportion of 97%. Throughout the fermentation process of kombucha, the fungal microbiota was primarily composed of yeast communities, including *Saccharomyces*, *Zygosaccharomyces*, *Pichia*, and *Dekkera*.

### 3.12. Correlation Between Microbiota and VFCs

**Figure 9:**
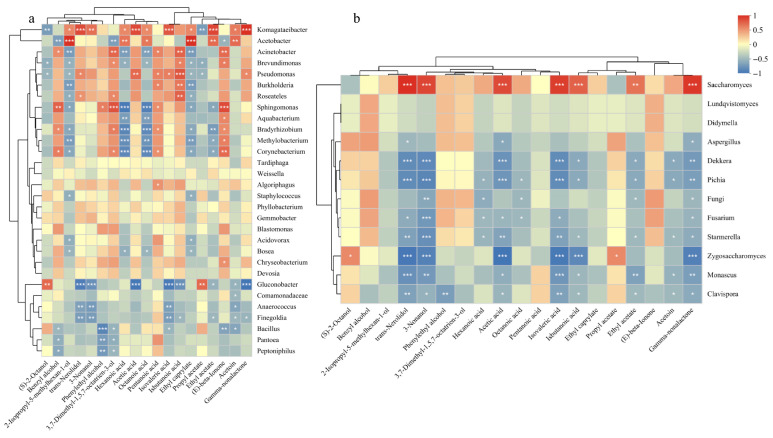
Spearman heatmap of VFCs and the bacterial and fungal communities in kombucha, showing the correlation between the bacterial community and VFCs at the genus level (**a**), and the correlation between the fungal community and VFCs at the genus level (**b**). *: *p* < 0.05, **: *p* < 0.01, ***: *p* < 0.001.

Changes in volatile compounds during fermentation may be associated with alterations in the microbial communities of the prepared kombucha samples. To analyze the correlation between microbiota and key VFCs during fermentation, we calculated the Spearman correlation coefficient [66]. As shown in Figure 9, a total of 11 VFCs exhibited significant positive correlation with *Komagataeibacter,* including trans-nerolidol, acetic acid, isovaleric acid, ethyl acetate, and γ-nonalactone, with correlation coefficients ranging from 0.72 to 0.90 (*p* < 0.05). *Komagataeibacter* predominates in the later stages of kombucha fermentation and serves as a representative of acetic acid bacteria, facilitating the production of compounds such as ethyl acetate, ethyl octanoate, and acetoin [67,68]. Isobutanoic acid showed a significant positive correlation with *Acinetobacter*, *Brevundimonas*, *Pseudomonas*, *Burkholderia*, and *Roseateles*. In addition, hexanoic acid and octanoic acid exhibited significant negative correlations with *Sphingomonas, Aquabacterium*, *Bradyrhizobium*, *Methylobacterium*, and *Corynebacterium*. *Sphingomonas* and *Gluconobacter* demonstrated significant positive correlations with characteristic flavor compounds of kombucha, including phenylethanol and 3,7-dimethyl-1,5,7-octatrien-3-ol, while showing significant negative correlations with hexanoic acid, acetic acid, octanoic acid, isovaleric acid, and isobutanoic acid (Figure 9a). Furthermore, benzyl alcohol, valeric acid, and (E)-β-ionone, which possess floral and fruity aromas, exhibited positive correlations with *Sphingomonas*, *Acinetobacter*, and *Corynebacterium*. As shown in Figure 9b, *Saccharomyces* showed significant positive correlations with trans-nerolidol, 3-nonanol, acetic acid, isovaleric acid, isobutanoic acid and γ-nonalactone, while *Zygosaccharomyces* was positively correlated with (S)-2-octanol and propyl acetate. In contrast, *Dekkera* and *Pichia* displayed negative correlations with trans-nerolidol, 3-nonanol, acetic acid, and isovaleric acid. *Dekkera* and *Pichia* are recognized as primary contributors to wine spoilage [69], and their overgrowth can lead to the production of high levels of acetic acid, acetates, and ketones, resulting in off-flavors and unpleasant odors in fermented foods [70]. However, the relationship between the microbiota and volatile compounds remains to be further verified.

## 4. Conclusions

This study systematically compares the physicochemical properties, bioactive potential, volatile flavor profiles, and microbial community dynamics of three types of kombucha (KBT, KBL, KRY) during fermentation at 28 °C, evaluating the feasibility of RYR as an alternative functional substrate for kombucha fermentation. The results indicate that the KRY sample exhibited the lowest pH value, the highest total acidity, and the most significant consumption of monosaccharides and total sugars, while forming a thicker bacterial cellulose membrane due to the accelerated proliferation of acetic acid bacteria and yeast. Furthermore, KRY demonstrated promising in vitro enzyme inhibitory activity related to hypoglycemic and hypolipidemic effects, whereas KBT maintained an irreplaceable antioxidant capacity, likely attributable to its natural tea polyphenol components. A total of 72 VFCs were identified, with 7 key aroma-active compounds significantly enriched in KRY, contributing to its highest sensory acceptance. Microbial community analysis revealed that the fermentation process led to a shift in dominant microbiota from the initial *Gluconobacter* to *Komagataeibacter* and *Acetobacter* as well as yeast post-fermentation. Moreover, RYR may promote the proliferation of functional groups positively correlated with key VFCs. In conclusion, RYR demonstrates significant potential as a functional substrate, providing a theoretical foundation for the development of KRY production. However, further experiments are necessary to validate the functional activity of kombucha in vivo and to confirm the association between flavor compounds and microbial dynamics.

## Figures and Tables

**Table 1:** Changes in Total Polyphenols and Total Flavonoids During Kombucha Fermentation.

Concentration (μg/mL)	Time (d)	KBT	KBL	KRY
Total Polyphenols	0	631.17 ± 4.16 ^Aa^	290.43 ± 1.15 ^Ba^	57.10 ± 2.14 ^Cc^
	3	448.33 ± 4.22 ^Ab^	241.97 ± 1.19 ^Bb^	67.23 ± 1.15 ^Cb^
	6	338.60 ± 2.00 ^Ac^	211.97 ± 1.53 ^Bd^	68.90 ± 0.01 ^Cb^
	9	318.40 ± 3.00 ^Ad^	237.93 ± 1.53 ^Bc^	80.67 ± 0.58 ^Ca^
Total Flavonoids	0	26.23 ± 0.50 ^Ad^	12.73 ± 0.06 ^Bd^	7.10 ± 0.01 ^Ca^
	3	35.47 ± 0.29 ^Aa^	28.63 ± 0.06 ^Ba^	6.30 ± 0.02 ^Cb^
	6	32.17 ± 0.64 ^Ac^	23.43 ± 0.74 ^Bb^	4.27 ± 0.06 ^Cc^
	9	34.33 ± 0.64 ^Ab^	20.01 ± 0.36 ^Bc^	3.93 ± 0.06 ^Cd^

^A–C^: Different capital letters indicate significant differences between groups. ^a–d^: Different lowercase letters indicate significant differences within groups.

**Table 2:** The equivalent scavenging capacity of kombuchas on DPPH and ABTS+.

VcE (mg/mL)	Time (d)	KBT	KBL	KRY
DPPH	0	1.2846 ± 0.0028 ^Aa^	1.1852 ± 0.0028 ^Be^	0.3500 ± 0.0029 ^Cd^
	3	1.2773 ± 0.0019 ^Aab^	1.2131 ± 0.0017 ^Bd^	0.3412 ±0.0033 ^Ce^
	6	1.2736 ± 0.0017 ^Ab^	1.2687 ± 0.0017 ^Aa^	0.4501 ± 0.0040 ^Bb^
	9	1.2809 ± 0.0006 ^Aab^	1.2523 ± 0.0006 ^Bc^	0.4299 ± 0.0028 ^Cc^
	12	1.2761 ± 0.0077 ^Ab^	1.2629 ± 0.0029 ^Bb^	0.4773 ± 0.0017 ^Ca^
ABTS+	0	2.1311 ± 0.0113 ^Aa^	1.3069 ± 0.01444 ^Bc^	0.4917 ± 0.01372 ^Ca^
	3	2.1102 ± 0.0026 ^Ab^	1.2069 ± 0.0637 ^Bd^	0.3870 ± 0.0026 ^Cd^
	6	2.0908 ± 0.0131 ^Ac^	1.4817 ± 0.02918 ^Ba^	0.4394 ± 0.0232 ^Cbc^
	9	2.0878 ± 0.0069 ^Ac^	1.3876 ± 0.0069 ^Bb^	0.4259 ± 0.0118 ^Cc^
	12	2.0757 ± 0.0001 ^Ac^	1.3788 ± 0.0207 ^Bb^	0.4573 ± 0.0119 ^Cb^

^A–C^: Different capital letters indicate significant differences between groups. ^a–d^: Different lowercase letters indicate significant differences within groups.

## Data Availability

All data supporting the findings of this study are included within the article and Supplementary Materials. Data will be made available on request.

## Supplementary Materials

The following supporting information can be downloaded at: https://www.mdpi.com/article/10.3390/foods15040747/s1, Figure S1: recipe instructions for preparing KBT, KBL, and KRY; Figure S2: comparisons based on VFCs including a PLS-DA score plot (a), permutation test plot (b), and the VIP primary values identified by the PLS-DA model (c); Figure S3: the permutation plots for KBT-0 vs. KBT-9, KBL-0 vs. KBL-9, KRY-0 vs. KRY-9, KBT-9 vs. KBL-9, KBL-9 vs. KRY-9, and KBT-9 vs. KRY-9 were shown in a, b, c, d, e, and f, respectively; Figure S4: orthogonal partial least squares regression analysis (OPLS-DA) used to analyze the data, with score plots comparing KBT-0 vs. KBT-9 (a), KBL-0 vs. KBL-9 (b), KRY-0 vs. KRY-9 (c), KBT-9 vs. KBL-9 (g), KBL-9 vs. KRY-9 (h), and KBT-9 vs. KRY-9 (i). The VIP primary values for these comparisons were shown in d, e, f, j, k, and l, respectively; Figure S5: Clustering heatmap illustrating the classification of 19 VFCs among KBT, KBL, and KRY; Table S1: Standard Curves for Total Polyphenols and Total Flavonoids; Table S2: Gas sensor array of E-nose; Table S3: VFCs detected in different kombuchas; Table S4: 19 Key Compound Aromas.
