# Supplementary figures and images for: Red Yeast Rice-Driven Kombucha Fermentation: A Novel Strategy for Developing Functional Beverages with Enhanced Hypoglycemic and Hypolipidemic Properties
Source: Foods. 2026 Feb 18;15(4):747. doi: 10.3390/foods15040747 (PMC12939368; doi:10.3390/foods15040747)

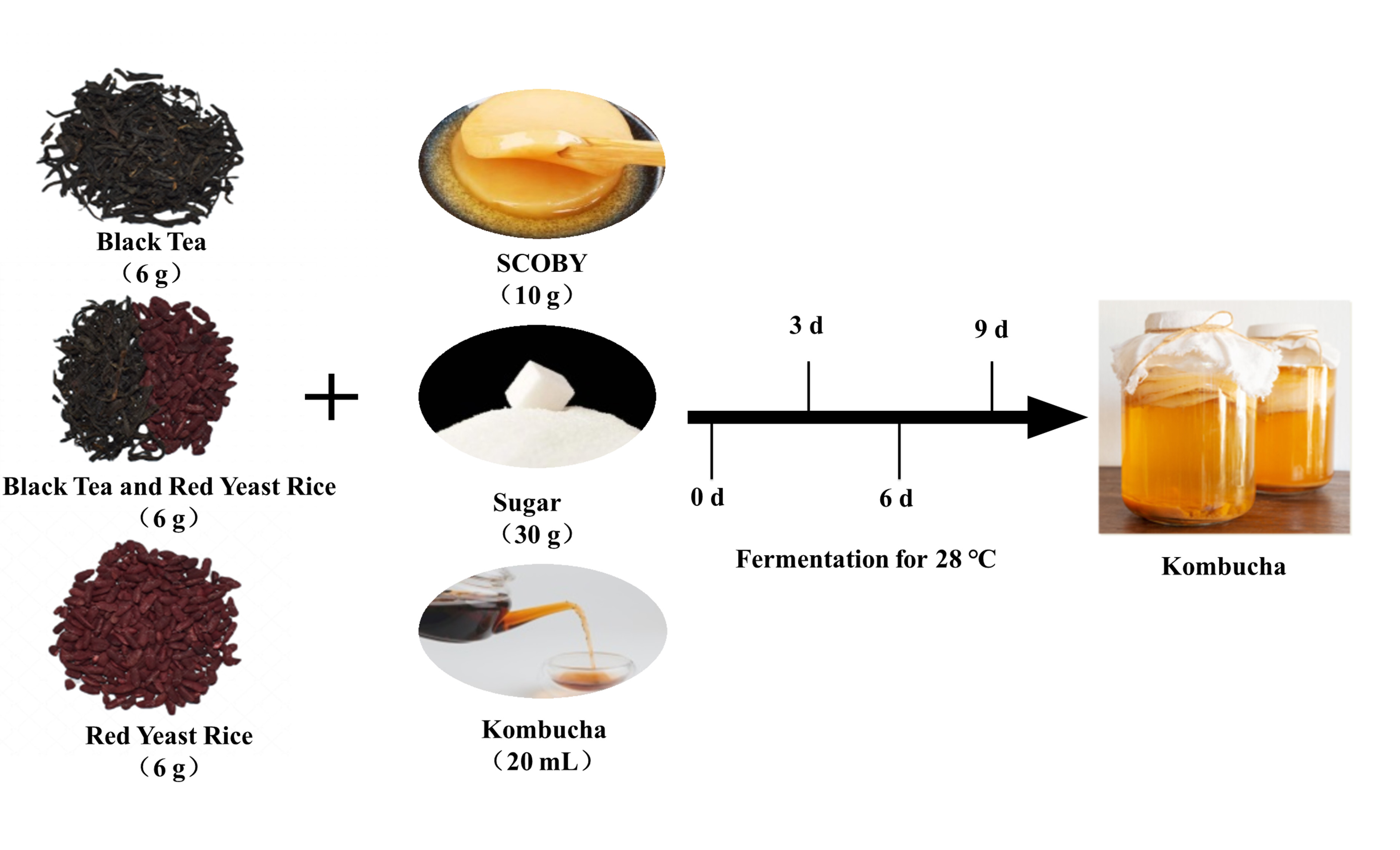

Supplement: Supplementary file 1 [file foods-15-00747-s001.zip › foods-4120328-supplementary/Fig.S1..jpg]

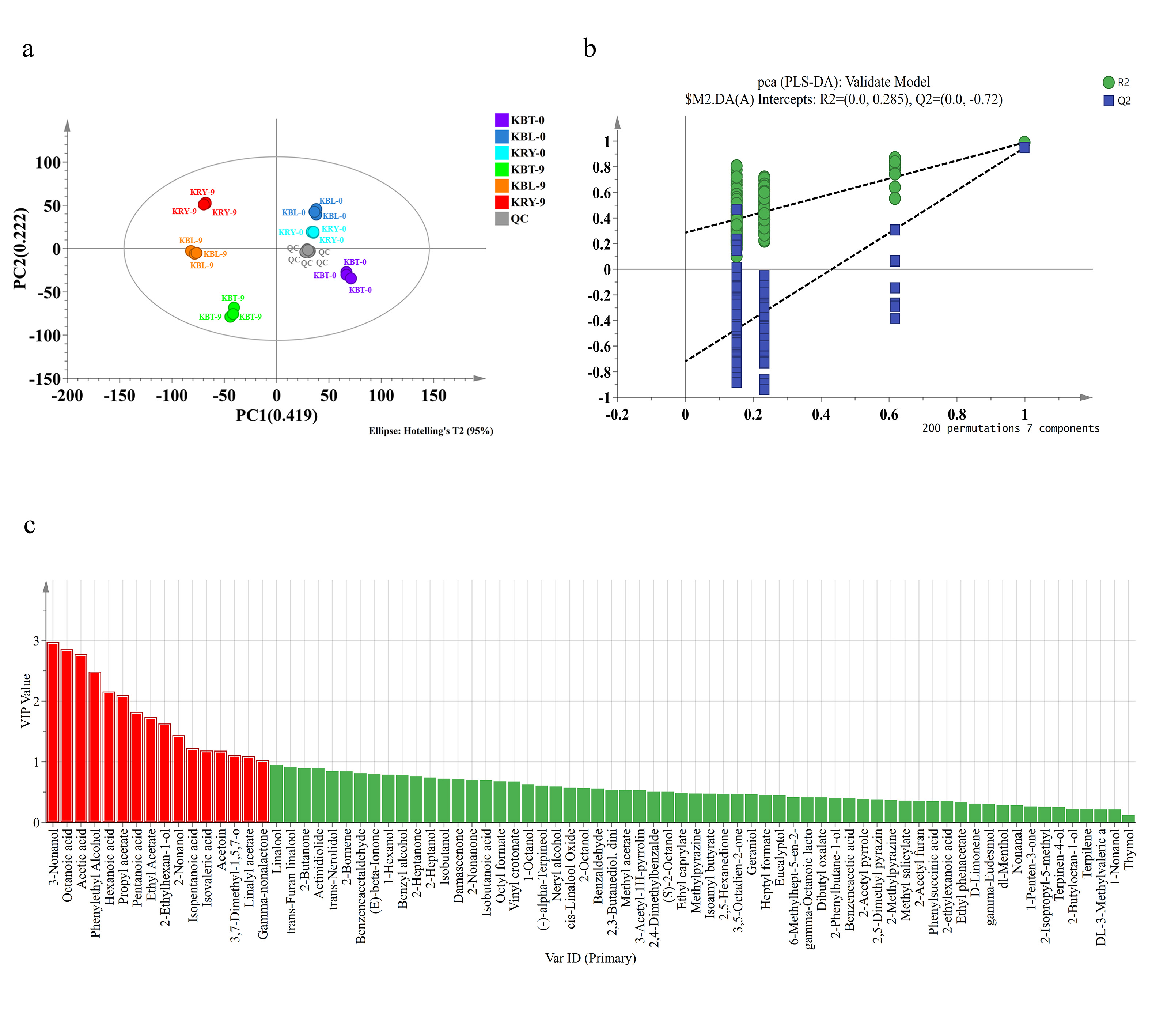

Supplement: Supplementary file 1 [file foods-15-00747-s001.zip › foods-4120328-supplementary/Fig.S2..jpg]

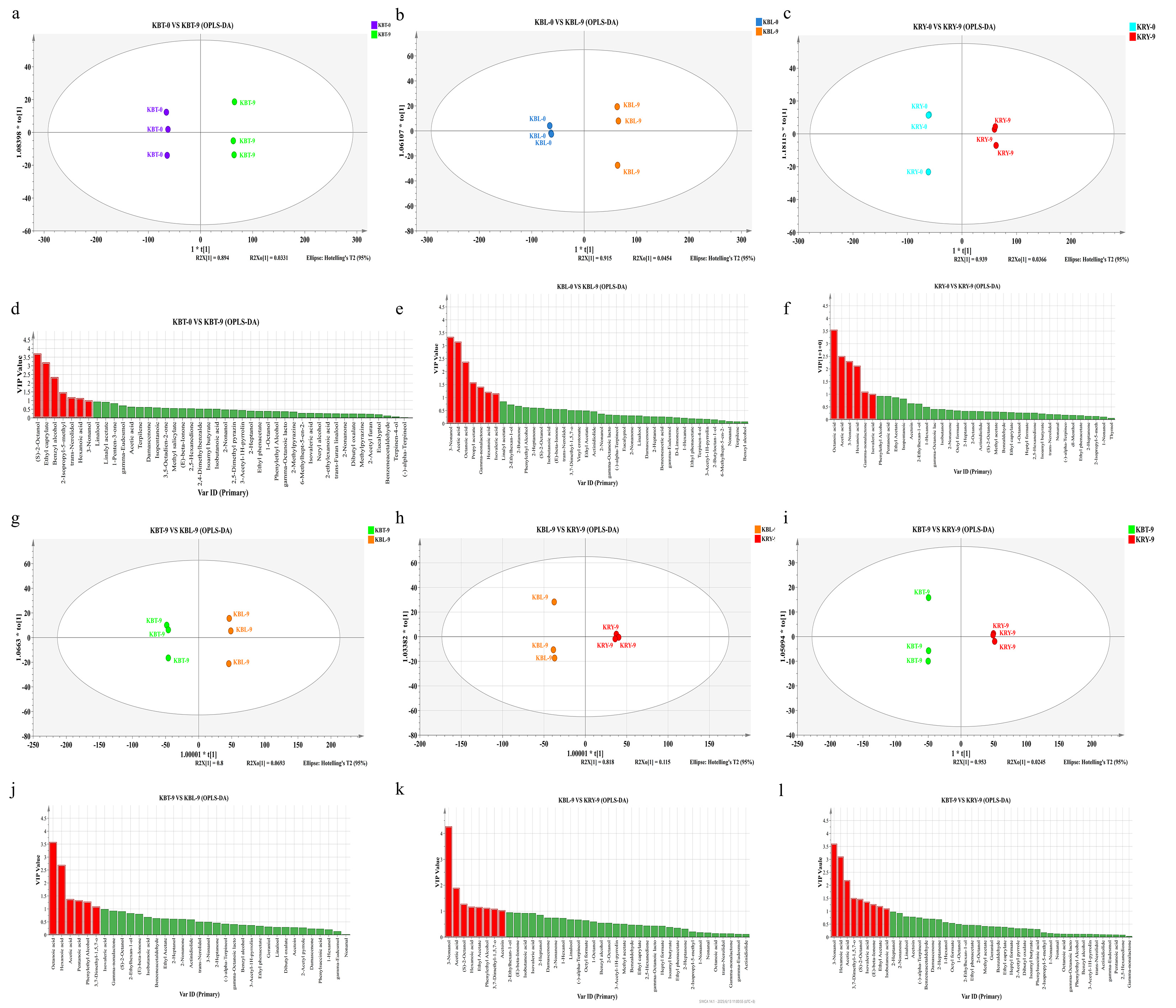

Supplement: Supplementary file 1 [file foods-15-00747-s001.zip › foods-4120328-supplementary/Fig.S3..jpg]

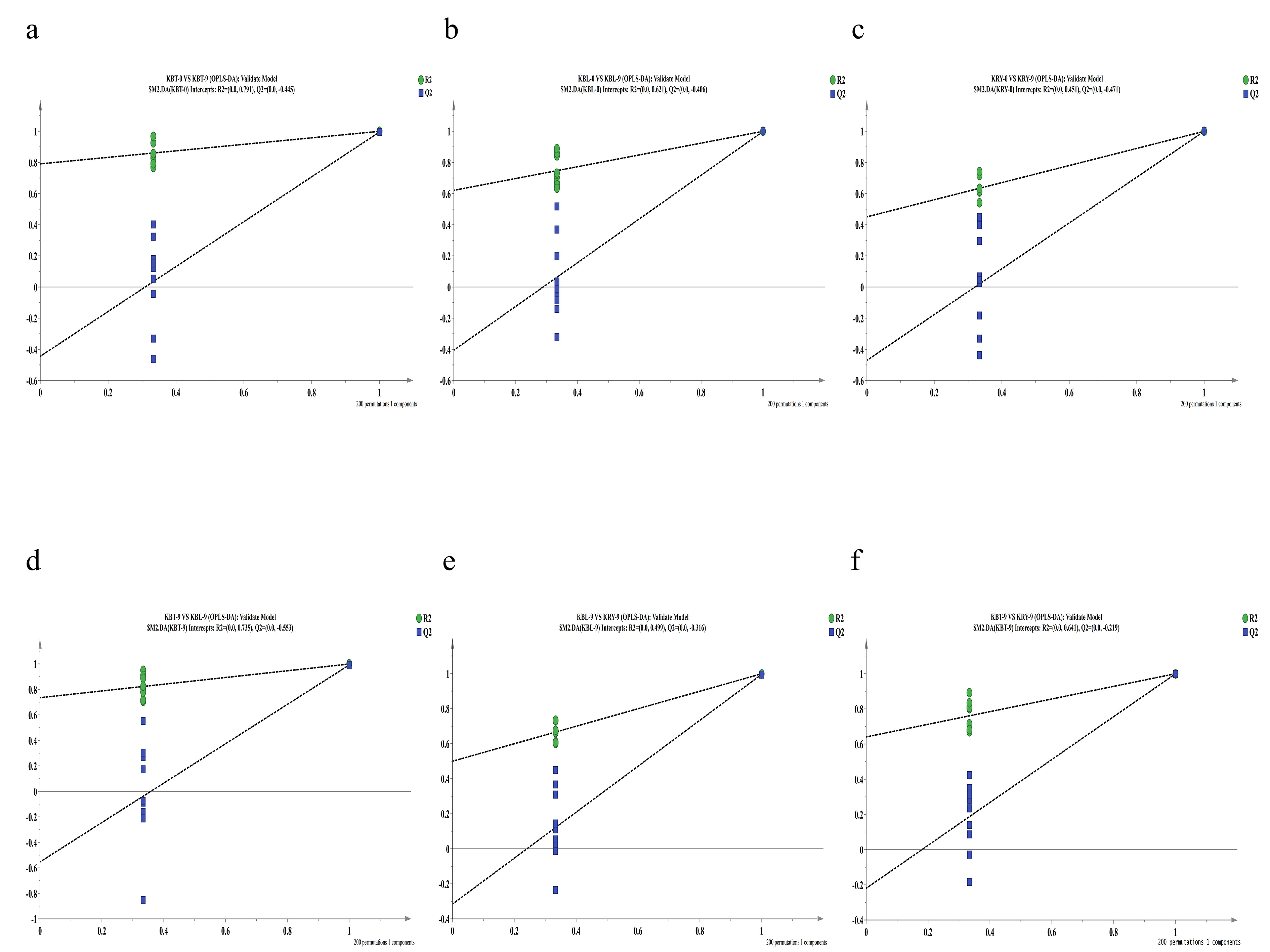

Supplement: Supplementary file 1 [file foods-15-00747-s001.zip › foods-4120328-supplementary/Fig.S4..jpg]

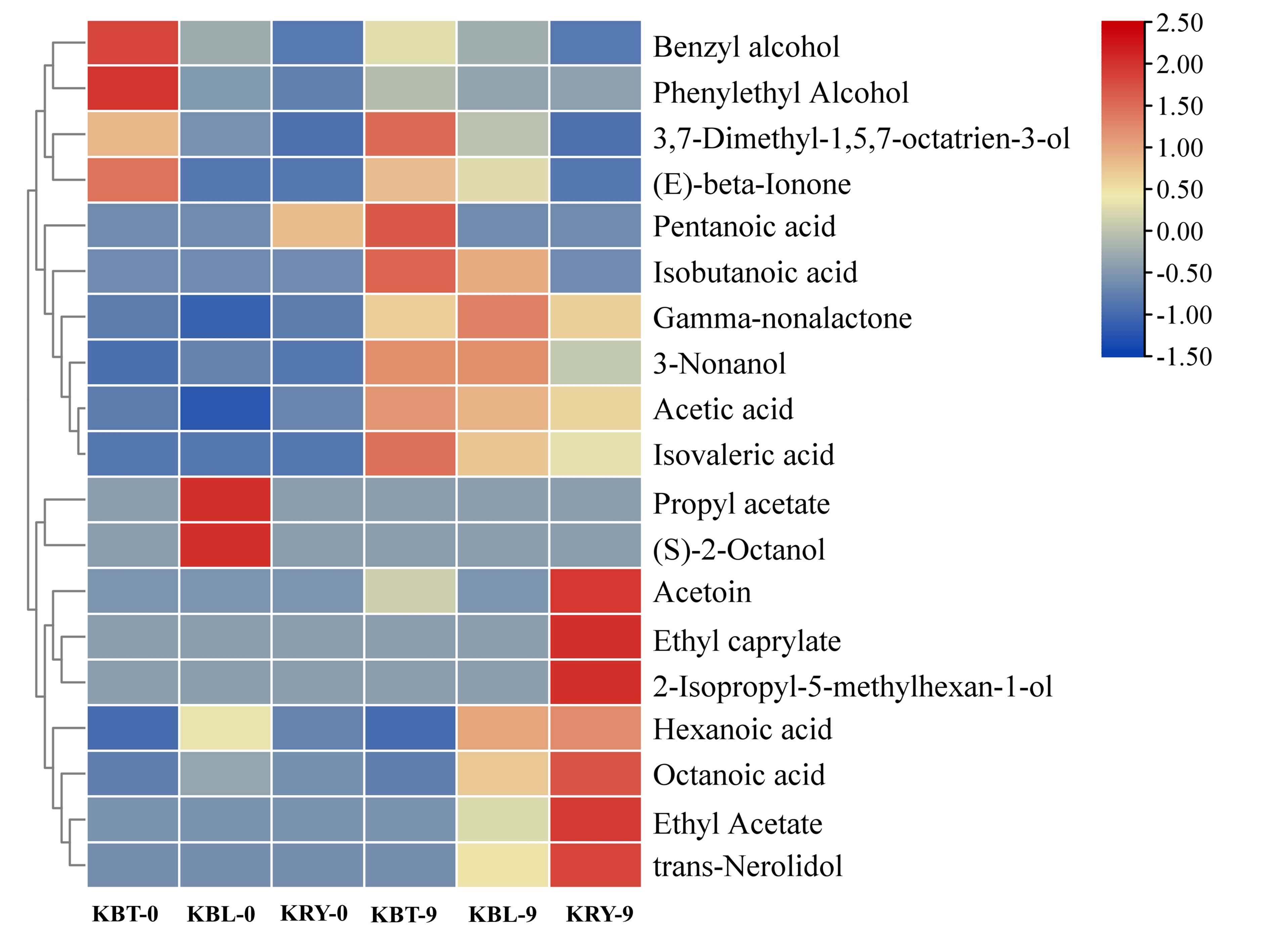

Supplement: Supplementary file 1 [file foods-15-00747-s001.zip › foods-4120328-supplementary/Fig.S5..jpg]
